# From disease to treatment: from rare skeletal disorders to treatments for osteoporosis

**DOI:** 10.1007/s12020-016-0888-7

**Published:** 2016-02-18

**Authors:** Natasha M. Appelman-Dijkstra, Socrates E. Papapoulos

**Affiliations:** Center for Bone Quality, Leiden University Medical Center, Albinusdreef 2, 2333 ZA Leiden, The Netherlands

**Keywords:** Osteoporosis, Sclerostin, Cathepsin K, Pycnodystostosis, Van Buchem disease, Sclerosteosis

## Abstract

During the past 15 years there has been an expansion of our knowledge of the cellular and molecular mechanisms regulating bone remodeling that identified new signaling pathways fundamental for bone renewal as well as previously unknown interactions between bone cells. Central for these developments have been studies of rare bone disorders. These findings, in turn, have led to new treatment paradigms for osteoporosis some of which are at late stages of clinical development. In this article, we review three rare skeletal disorders with case descriptions, pycnodysostosis and the craniotubular hyperostoses sclerosteosis and van Buchem disease that led to the development of cathepsin K and sclerostin inhibitors, respectively, for the treatment of osteoporosis.

## Introduction

Osteoporosis is characterized by the imbalance between bone resorption by osteoclasts and bone formation by osteoblasts that leads to loss and structural decay of bone and, consequently, reduced bone strength and increased fragility. This disturbance of bone remodeling provides the rationale for the development of pharmacological agents to prevent bone loss and reduce the risk of fractures in patients with osteoporosis. The majority of currently available treatments reduce bone resorption while only PTH peptides stimulate bone formation. Although these treatments have greatly improved the management of patients with osteoporosis, they do not eliminate fracture risk, have rather limited effect on the risk of nonvertebral fractures, and they cannot build new bone at all sites important for bone strength.

During the past 15 years there has been an expansion of our knowledge of the cellular and molecular mechanisms regulating bone remodeling that identified new signaling pathways fundamental for bone renewal as well as previously unknown interactions between bone cells. Central for these developments have been studies of rare bone disorders. For example, the finding that loss-of-function mutations of LRP5 cause the osteoporosis-pseudoglioma syndrome while gain-of-function mutations of this receptor cause the High Bone Mass phenotype revealed for the first time the importance of the Wnt signaling pathway in bone formation [1–3]. Moreover, study of patients with osteopetrosis provided evidence that bone resorption and bone formation are not necessarily coupled if osteoclasts, despite losing their functional ability, remain intact [4]. These findings, in turn, have led to new treatment paradigms for osteoporosis some of which are at late stages of clinical development. These new treatments have been recently reviewed [5–7].

The relevant clinical question is whether, apart from providing new treatment targets, study of patients with rare skeletal disorders can improve our understanding of the effects of new treatments on bone metabolism. In this article, we address this question and we discuss three bone dysplasias pycnodysostosis and the craniotubular hyperostoses sclerosteosis and van Buchem disease, including case descriptions, that led to the development of cathepsin K and sclerostin inhibitors, respectively, for the treatment of osteoporosis.

## Pycnodysostosis

**Fig. 1:**
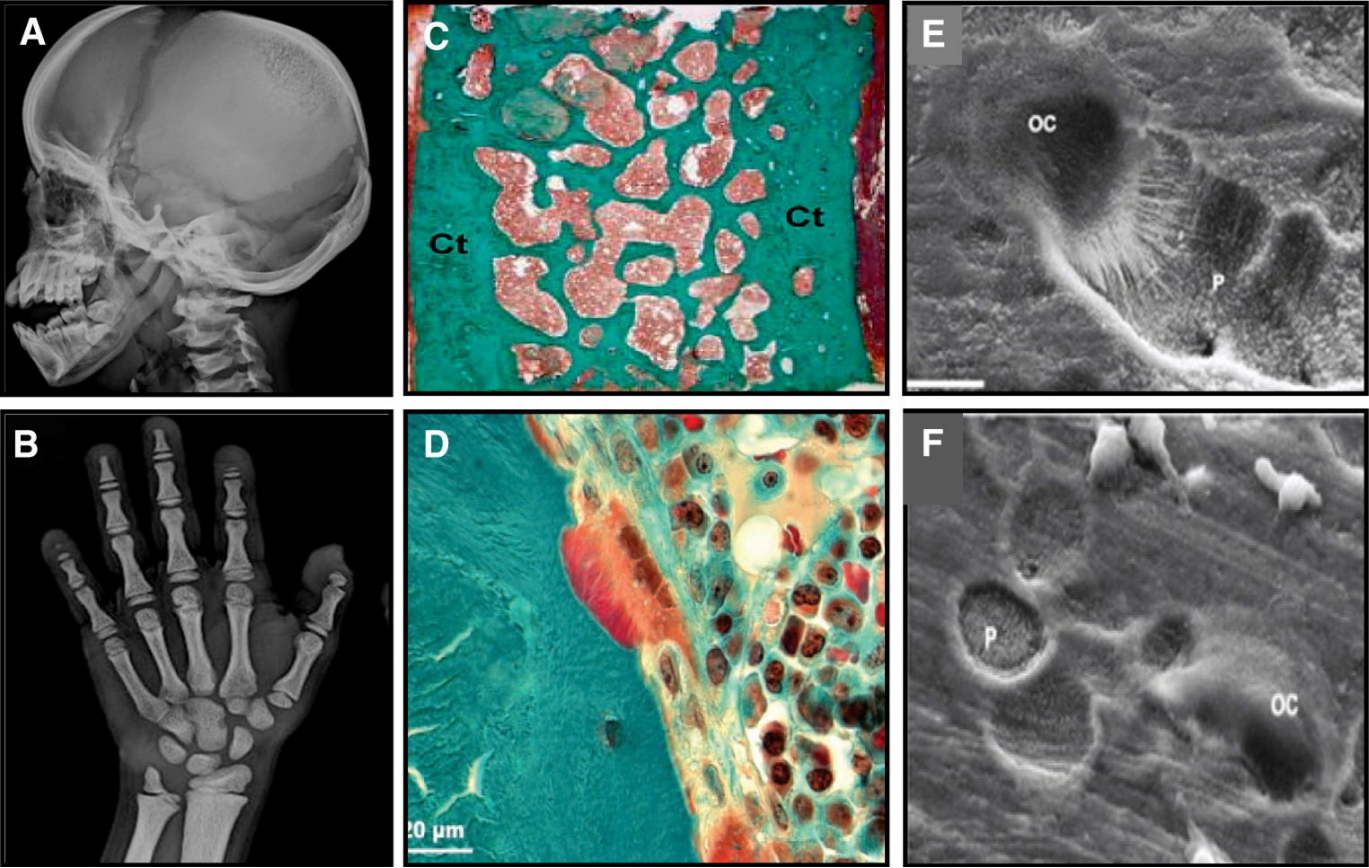
Pycnodysostosis: **a** Open sutures; **b** Acro-osteolysis; **c** Iliac crest bone biopsy showing cortical (Ct) and trabecular osteosclerosis (from [11]); **d** High magnification of an iliac crest bone biopsy showing an osteoclast adjacent to a resorption lacuna filled with unmineralized matrix (from [12]); SEM images of odanacatib-treated osteoclast culture on dentine slides: **e** Control, showing a deep resorption pit; **f** Treated, showing discrete, small, shallow resorption pits (from [87])

Pycnodystostosis (OMIM 265800), meaning dense defective bone, is a rare, autosomal recessive osteochondrodysplasia. The first case was described in 1923 but the features of the disease were defined by Maroteux and Lamy in 1962 [8, 9]. The prevalence of pycnodysostosis is estimated to be 1–1.7 per million, it is equally distributed between women and men and about 200 cases have been reported in the literature [10]. The disease is characterized by osteosclerosis, short stature, acro-osteolysis of the distal phalanges, clavicular dysplasia, skull deformities with delayed suture closure, and bone fragility (Fig. 1a, b). The most commonly described phenotype of pycnodystostosis is short stature with increased bone mineral density and increased bone fragility. In infants, open fontanels and sutures with frontal and parietal bossing and hypoplasia of the maxilla and mandible with an obtuse mandibular angle are frequently seen. Dentogenesis with delayed eruption of the teeth may also be present.

In a limited number of adequately evaluated bone biopsies from patients with pycnodysostosis there was cortical and trabecular osteosclerosis with increased cortical width, findings consistent with the high values of BMD of affected patients [11, 12]. Eroded surfaces were normal or slightly increased with adjacent multinucleated osteoclasts but resorption lacunae were not deep and contained unmineralized bone matrix (Fig. 1c, d). These observations indicate dysfunction of osteoclasts that are unable to digest the collagenous bone matrix after the dissolution of the mineral in the resorption space. Bone formation was decreased but osteoid thickness was low, normal, or increased possibly due to differences in the age of the studied patients. Tetracycline labels were decreased but present in bone biopsies. Lamellar organization was disturbed to varying degrees and mineralized cartilage residuals were observed to a lesser extent than in cases of osteopetrosis. It was suggested that these disturbances of the quality of bone matrix may contribute to bone fragility in pycnodysostosis [12].

### The cause

In 1995, Gelb and colleagues performed a genome-wide search in a large consanguineous Israeli Arab family with 16 affected individuals, identified the locus of pycnodysostosis to chromosome 1q21, and found a mutation in the gene encoding cathepsin K by positional cloning [13]. These authors also provided further evidence that cathepsin K deficiency causes pycnodysostosis by finding additional mutations in two unrelated Mexican and American-Hispanic families. Notably, these observations coincided with the elucidation of the localization of cathepsin K in osteoclasts and its action in bone resorption [14–16]. Further studies of patients with pycnodysostosis confirmed these original findings and revealed the presence of at least 35 different mutations in the cathepsin K gene with no, however, clear genotype-phenotype associations.

Cathepsin K, a member of a family of cysteine proteases with shared sequence and structural homology, is responsible for the digestion of the organic matrix of bone [17]. It is synthesized as a pro-enzyme before being transported to lysosomes where it is cleaved to produce the active enzyme. Cathepsin K is abundantly expressed in osteoclasts, but has also been detected in macrophages and bone marrow-derived dendritic cells, but hardly in splenic T cells [18]. In mature osteoclasts, cathepsin K is essential for osteoclast-mediated bone resorption because it degrades collagen type I and other bone matrix proteins [19–21]. The enzyme cleaves the N-telopeptide, generating NTX and degrades C-terminal telopeptide of type I collagen (1CTP), producing CTX [17]. In line with these actions of cathepsin K and consistent with the histological findings, patients with pycnodysostosis have normal serum TRAP5b values, a marker of osteoclast number, while serum NTX and CTX values are low and those of serum 1CTP are increased [22].

Mice deficient in cathepsin K had osteosclerosis in the presence of fully differentiated osteoclasts while mice overexpressing cathepsin K had decreased trabecular bone volume and increased bone turnover [23, 24]. Bone biopsies from cathepsin K-deficient mice confirmed the decreased bone resorption and revealed the presence of increased osteoclast numbers with maintenance, however, or increase in bone formation [25]. To elucidate the mechanism of action of cathepsin K in bone remodeling, particularly in bone formation, the enzyme was deleted in hematopoietic cells or specifically in osteoclasts and cells of the monocyte-osteoclast lineage and resulted in increased bone volume. This increase in bone volume was accompanied by an increase in bone formation rates as well as in osteoclast and osteoblast numbers [26]. However, in the same study, deletion of cathepsin K in osteoblasts had no effect on bone turnover or bone formation rates [26], demonstrating that the increase in bone formation was driven by the osteoclasts. These observations confirmed that in the absence of cathepsin K, bone resorption and formation are not coupled and that there are signals from osteoclasts to the osteoblast lineage (e.g. SP1) that maintain bone formation in the presence of decreased bone resorption. In addition, matrix-derived factors that can stimulate bone formation (e.g. IGF1) and are not degraded due to the lack of cathepsin K may be contributory [27].

### The patient

A 34-year-old female of Indian origin was investigated for the first time at the age of 11 years for delayed growth and at the age of 15 years the diagnosis of osteopetrosis was made by a pediatric endocrinologist. Parents were consanguineous, she had a brother and a sister who were healthy. She was seen for the first time in our institution at the age of 16 years. She had no specific complaints, no vision or hearing problems and had never sustained a fracture. At the age of 19, she had an adjustment operation of the maxilla to improve its form and her eating. Her height was 140.5 cm, weight 35 kg, blood pressure 100/55 mm Hg, and pulse rate 76/min. She had micrognathia but clinical examination was otherwise unremarkable and she had no hepatosplenomegaly. Serum 25-hydroxyvitamin D was low (25 nmol/l) with a slightly raised plasma PTH (9.5 pmol/l); both these abnormalities were corrected with vitamin D supplementation. Serum and urinary calcium excretion and serum phosphate were always within the normal range. Serum alkaline phosphatase was always normal for age: 146 U/l at 16 years, 102 U/l at 19 years, 109 U/l at 20 years, 69 U/l at 23 years, and 69 U/l at 25 years (normal adult range 40–120 U/l). In addition, serum P1NP, another marker of bone formation, was 68 ng/ml (upper limit of normal range) at the age of 25 years. BMD was increased (femoral neck* Z*-score +3.3, lumbar spine *Z*-score +1.2) but did not increase further with time, and skeletal radiographs showed diffuse osteosclerosis, mandibular hypoplasia, and mild acro-osteolysis of the phalanges. DNA testing revealed a homozygous missense mutation of the *CTSK* gene (exon 6 p.Ile249Thr). Histomorphometry of a transiliac bone biopsy was consistent with previous reports.

This patient with cathepsin K deficiency had typical phenotypic, radiographic, and histological features of pycnodysostosis but she had never experienced a fragility fracture. Increased bone fragility is always mentioned as a typical feature of pycnodysostosis. However, this appears not to be the case. In a detailed description of 16 patients from different families with 8 different mutations, all had short stature, 14/16 had acro-osteolysis while 8/16 had fractures as was also reported in a smaller study [10, 28]. In a review of published cases, fractures were reported in 67 % of patients [29]. The reason for the lack of bone fragility in our patient is not clear and does not appear to be directly related to the changes observed in bone biopsy. Furthermore, bone formation markers were always normal for age over a period of 11 years, consistent with reports of normal values of biochemical markers of bone formation in patients with pycnodystostosis [22, 30].

### Cathepsin K inhibitors

The discovery that in cathepsin K deficiency, contrary to treatments with antiresorptive medications (e.g. bisphosphonates, denosumab), the decrease of bone resorption was associated with ongoing bone formation supported the development of a new class of antiresorptive agents targeting cathepsin K [31].

#### Animal studies

A number of cathepsin K inhibitors were synthesized and were tested in preclinical studies. In vitro studies showed inhibition of osteoclastic bone resorption and formation of shallow resorption pits (Fig. 1e, f). However, rodents that are used extensively in the development of antiosteoporotic drugs could not be used in in vivo preclinical studies of cathepsin K inhibitors due to differences in amino acid sequence between rodent and human cathepsin K. Most of the preclinical studies with cathepsin K inhibitors were performed in primates. In addition, a rabbit model, which, in contrast to rodents, undergoes cortical Haversian remodeling, was used in several studies. In OVX primates, cathepsin K inhibitors act differently from bisphosphonates and denosumab. Whereas treatment with relacatib, odanacatib, or ONO-5334 reduced bone resorption in OVX monkeys, it also increased the number of non-resorbing osteoclast at the bone surfaces and, depending on the bone envelope, decreased, maintained, or even increased bone formation [32–34]. For example, odanacatib treatment reduced trabecular and intracortical bone formation while it preserved endocortical bone formation and increased periosteal bone formation in the femoral neck, proximal femur, and central femur; the latter effect was also observed in the mid-shaft femur of OVX monkeys treated with balicatib [35, 36]. These changes were associated with increases in volumetric BMD of both trabecular and cortical bone and increases in cortical area of the femoral neck and cortical thickness of the proximal tibia. Importantly, the increases in bone mass were positively and significantly related with bone strength. The mechanism(s) responsible for the site-specific effect of cathepsin K inhibitors on bone formation has not yet been elucidated and its relevance in humans remains to be established [37]. Interestingly, excess periosteal bone formation over resorption, possibly supernormal, was reported in a detailed rib biopsy from a patient with pycnodystostosis [38].

In OVX rabbits, odanacatib reduced bone resorption, preserved bone formation in trabecular and endocortical surfaces, increased hip BMD dose-dependently, and improved biomechanical properties of the vertebrae and the central femur [39, 40]. Furthermore, odanacatib did not impair callus formation or its biomechanical integrity in a rabbit model of fracture healing [41]. A recent study reported that while odanacatib restored trabecular BMD, microstructure and biomechanical properties, and increased bone formation and cortical thickness of the central femur in osteopenic rabbits, it also led to loss of crystal heterogeneity that appeared to contribute to cortical brittleness [42]. The latter report, that contrasts all other observations, needs to be confirmed in further studies.

#### Human studies

Clinical testing of the earlier developed cathepsin K inhibitors relacatib and balicatib was interrupted because of lack of specificity for cathepsin K (relacatib) or accumulation in lysosomes of cells other than the osteoclasts (lysosomotropism) leading to off-target effects (balicatib). Odanacatib and ONO-533, however, did not show any evidence of off-target effects in initial clinical studies [43–45].

The efficacy and safety of ONO-5334 and odanacatib were assessed in phase 2 clinical trials in postmenopausal women with low bone mass. Both cathepsin K inhibitors were administered orally, without regard to food intake, either daily (ONO-5334) or once-weekly (odanacatib). Adverse, off-target, effects such as morphea-like lesions and infections, previously observed with balicatib treatment, were not observed in either of these clinical trials. Two-year treatment with ONO-5334, 300 mg per day, reduced urinary NTX/Cr by 66 % while serum BASP levels returned to baseline following an initial decline of 13 % [45]. In addition, ONO-5443, contrary to alendronate, did not reduce serum TRAP5b levels. BMD of the spine and hip increased by 6.7 and 3.4 %, respectively.

**Fig. 2:**
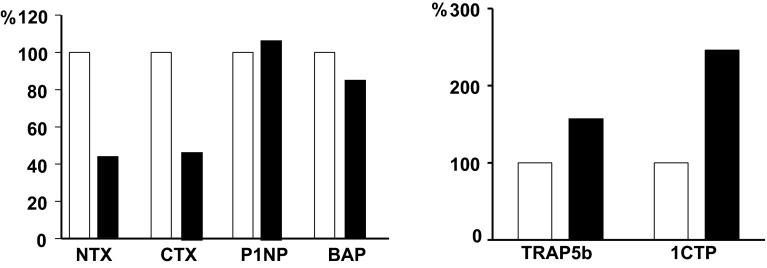
Percent changes of biochemical markers in serum of women treated with odanacatib 50 mg once weekly for 5 years. *Open bars* baseline; *Closed bars* 5 years (from [5])

A two-year, phase 2 dose-finding clinical trial of odanacatib was extended for an additional 3 years [43, 44]. Compared with baseline, 5-year treatment with odanacatib 50 mg once-weekly reduced biochemical markers of bone resorption by about 55 % while markers of bone formation after an initial decrease returned to baseline values. As expected by the mechanism of action of the inhibitor, serum levels of TRAP5b and 1CTP increased (Fig. 2) [45]. Odanacatib treatment was associated with continuous increases in BMD by 11.9, 8.5, and 9.8 % at the spine, total hip, and femoral neck, respectively. Odanacatib given for 2 years was further shown to increase volumetric BMD and estimated bone strength at both the hip and the spine in postmenopausal women [46–48]. Rates of adverse events were similar between placebo- and odanacatib-treated women. The action of odanacatib on BMD and bone turnover markers was reversible. Following discontinuation of odanacatib, there was a transient rebound of the levels of biochemical markers of bone turnover followed by decreases of BMD to baseline values; a response different from that following discontinuation of bisphosphonate treatment but similar to that after discontinuation of denosumab.

A phase 3 placebo-controlled, event-driven clinical trial with a preplanned extension was designed to assess the anti-fracture efficacy and safety of odanacatib in postmenopausal osteoporosis (LOFT trial) [49]. In total, 16,713 women with osteoporosis were randomized to receive placebo or odanacatib 50 mg one-weekly. In July 2012, the study was terminated on the recommendation of an independent Data Monitoring Committee (DMC) because of efficacy and a favorable benefit/risk profile of odanacatib relative to placebo. The DMC also recommended that additional safety data should be obtained in the preplanned, blinded extension study. Compared with placebo, treatment with odanacatib decreased significantly the incidence of new and worsening morphometric vertebral fractures by 54 %, of hip fractures by 47 %, of non-vertebral fractures by 23 %, and of clinical fractures by 72 % [50]. Odanacatib treatment led to progressive increases in BMD at lumbar spine and total hip: 11.2 % and 9.5 %, respectively, versus placebo over 5 years. Adverse events were generally well balanced between groups. Adjudicated morphea-like skin lesions occurred more frequently in odanacatib-treated patients (*n* = 12) vs placebo (*n* = 3) and resolved/improved after study drug discontinuation. Adjudicated femoral shaft fractures with atypical features occurred only in odanacatib-treated patients (*n* = 5) while no cases of ONJ were reported. No meaningful differences between groups were observed in adjudicated systemic sclerosis, respiratory infections, or delayed fracture union. Major cardiovascular events were generally balanced; however, there were numerically more adjudicated strokes with ODN than with placebo; final blinded adjudication of major cardiovascular events is ongoing [50].

### Comment

Deficiency of cathepsin K in humans, as it occurs in pycnodysostosis, is associated with high BMD but also increased bone fragility in about half of the patients. On the other hand, inhibition of cathepsin K in animal models increased bone mass and strength, and initial results of human studies demonstrated increases in BMD associated with significant reduction of fracture risk. Thus, while the human disease provided an excellent model for the identification of cathepsin K and its importance in bone resorption could not fully predict the effects of cathepsin K inhibition in humans with osteoporosis. In interpreting these findings it is important to differentiate the life-long complete and permanent effect of cathepsin K deficiency on the skeleton as opposed to its short-term, transient, and reversible inhibition in subjects with low bone mass. In addition, lack of a skeletal phenotype in heterozygote carriers of pycnodysostosis indicates that the degree of life-long inhibition of cathepsin K may be important. Results of the long-term effects of treatment of humans with cathepsin K inhibitors will help to fully clarify this issue.

## Sclerosteosis

**Fig. 3:**
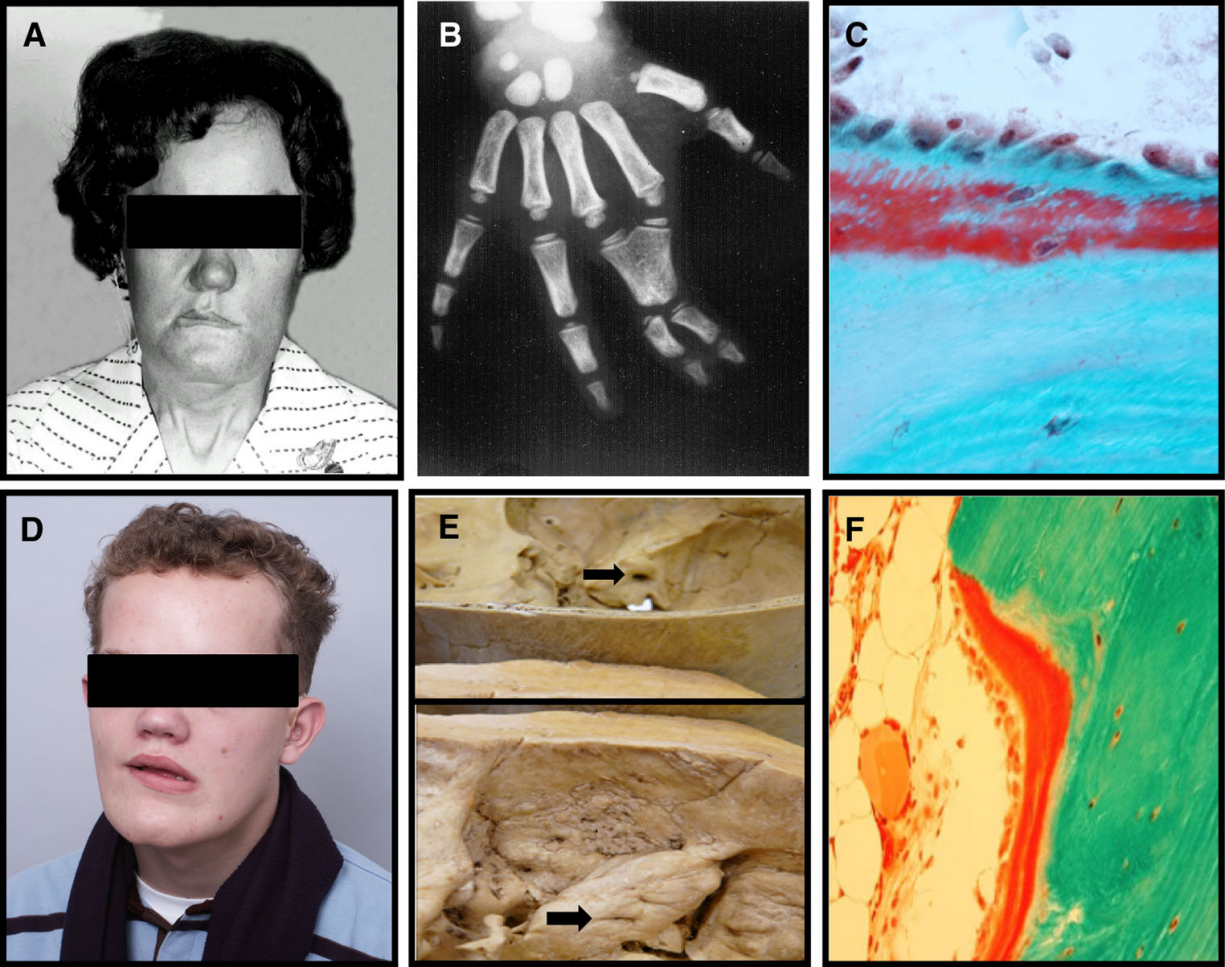
*Sclerosteosis*: **a** Enlarged skull and mandible with facial palsy; **b** Syndactyly; **c** Biopsy of compact bone with high numbers of osteoblasts and osteoid *van Buchem disease*: **d** Typical features with facial palsy; **e** Petrous part of temporal bone and acoustic meatus (*arrows*) of a normal skull (*upper*) and of a skull of a patient (*lower*); note the increased thickening and the narrowing of the meatus (from [88]); **f** Increased bone formation in a biopsy from compact bone

Sclerosteosis (OMIM 269500) is a very rare, autosomal recessive bone sclerosing dysplasia belonging to the group of craniotubular hyperostoses. It was first described as “osteopetrosis with syndactyly” by Truswell in 1958 and the term osteosclerosis was coined by Hansen in 1967 [51, 52]. About 100 cases have been reported in the literature, mainly in members of the Afrikaner community in South Africa. In this population, the estimated carrier rate is high being 1 in 140 individuals. The disease is characterized by bone overgrowth and generalized osteosclerosis. Affected patients are tall for age, and clinical manifestations are most pronounced in the mandible and the skull with characteristic enlargement of the jaw and facial bones leading to facial distortion and cranial nerve deficits such as hearing loss (100 %) and facial palsy (89 %), and less frequently loss of vision or smell (Fig. 3a). The most severe and life-threatening complication of sclerosteosis is increased intracranial pressure mainly as a result of decreased intracranial volume due to the thickening of the calvaria and the skull base. In the past, this has been a common cause of sudden death of patients with sclerosteosis. Patients with sclerosteosis have, in addition, digit malformation such as syndactyly (52 %) and nail hypoplasia (63 %) (Fig. 3b). The disease is progressive but clinical signs and symptoms stabilize after the third decade of life [53]. It is noteworthy, that apart from the characteristic skeletal changes, the general health of patients with sclerosteosis is otherwise good.

Skeletal radiographs show dense bones and cortical hyperostosis resulting from increased endosteal thickening of tubular bones. These changes are reflected in greatly increased BMD values at the hip and the spine with* Z* scores sometimes exceeding +10 [54]. In contrast to osteopetrosis, fractures have never been reported in patients with sclerosteosis [53, 55]. In a small number of bone biopsies obtained from patients with sclerosteosis, bone formation was greatly increased in trabecular and cortical bone (Fig. 3c) [53, 56]. The newly laid bone was lamellar with no mineralization defect; data on bone resorption were variable with decrease or no change in bone resorption. Bone material composition evaluated in surgically obtained specimens of compact bone showed reduction of bone matrix mineralization with increased heterogeneity of mineralization [57].

In patients with sclerosteosis, biochemical markers of bone formation show a normal, age-related pattern, increasing during childhood and adolescence to levels, however, higher than in healthy controls and declining after the completion of the growth spurt to levels around the upper limit of the normal adult range [53]. Biochemical markers of bone resorption follow a similar pattern but remain within their respective normal values. No abnormalities have been reported in serum calcium, phosphate, and PTH concentrations.

### The cause

**Fig. 4:**
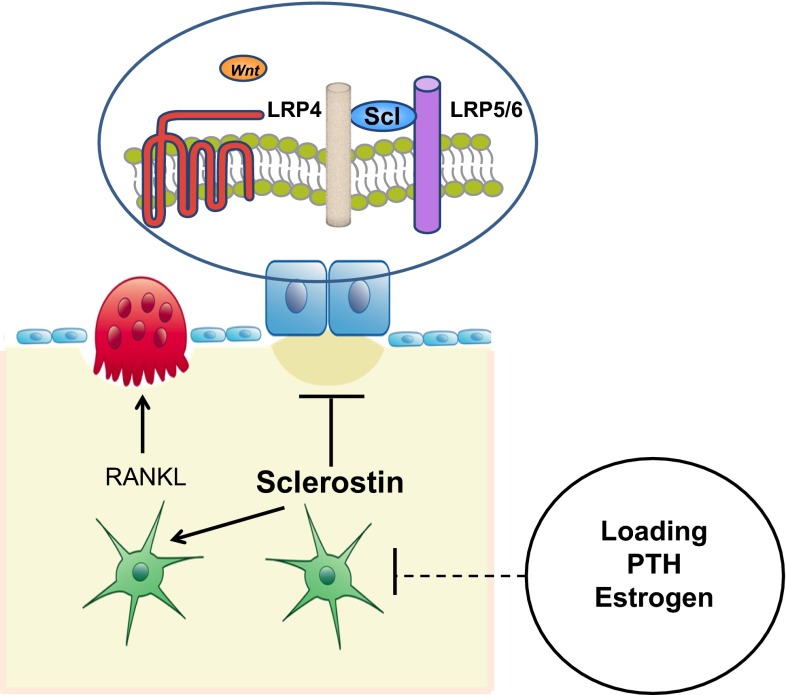
Schematic representation of sclerostin actions. Osteocyte-produced sclerostin inhibits the proliferation, differentiation and survival of osteoblasts and reduces bone formation; it stimulates also the production of RANKL by neighboring osteocytes and bone resorption. In osteoblasts, sclerostin binds to LRP5/6 and inhibits the Wnt signaling pathway, an action facilitated by LRP4. Production of sclerostin is decreased by mechanical loading, PTH, estrogens and other factors (from [67])

In 2001 two groups independently identified mutations in a new, rather small gene named *SOST* located on chromosome 17q12-21 that encodes for the protein sclerostin [58, 59]. At least 8 different mutations of the *SOST* gene have been reported leading to loss-of-function of sclerostin. Sclerostin is a glycoprotein with a cystine knot and three loops that is synthesized in the skeleton exclusively by mature osteocytes and inhibits bone formation at the bone surface by antagonizing the Wnt signaling pathway [60, 61]. Sclerostin binds to the first propeller of the LRP5/6 receptor and disables the formation of the co-receptor complex between LRP5/6 and the frizzled receptor. The action of sclerostin on the Wnt signaling pathway is facilitated by LRP4 [62, 63]. *SOST* mRNA is expressed in many tissues, especially during embryogenesis, but sclerostin is expressed postnatally only in terminally differentiated cells embedded within a mineralized matrix (osteocytes, mineralized chrondocytes and cementocytes). Consistent with this restricted expression of sclerostin, patients with sclerosteosis have no renal or cardiovascular abnormalities. Sclerostin in addition to its action on bone formation, stimulates the production of RANKL from neighboring osteocytes and increases bone resorption [64–66]. The production of sclerostin is regulated by different factors the most important being mechanical loading, PTH and estrogens, all of which reduce the production of sclerostin by osteocytes (Fig. 4) [67]. As expected, sclerostin was not expressed in bone biopsies from patients with sclerosteosis.

Targeted deletion of the *Sost* gene in mice increased bone mineral density at all skeletal sites and bone strength while mice overexpressing *Sost* were osteopenic [68, 69]. BMD increased progressively from 1 to 4 months of age, continuously but at a slower rate between 4 and 12 months and maintained a peak up to 18 months. MicroCT and histological analyses showed doubling of trabecular bone volume and thickness of the distal femur and of the cortical area of the femoral shaft due to increased rate of bone formation at all skeletal envelopes (trabecular, endocortical and periosteal) while osteoclast surface was not different from that of wild-type animals [68]. Similar to humans, bone matrix mineralization was reduced in sclerostin deficient mice [57]. There was, thus, a remarkable concordance of the findings in humans and mice with sclerostin deficiency.

## Van Buchem disease

The disease (OMIM 239100) was first described by van Buchem et al. in 1955 as “hyperostosis corticalis generalizata familiaris” [70]. It is a very rare, autosomal recessive craniotubular hyperostosis phenotypically very similar to sclerosteosis (Fig. 3d). There are about 30 known cases, the vast majority inhabitants of a small fishing village in the Netherlands. The carrier rate of the disease is unknown. Clinical manifestations of 15, recently evaluated patients with van Buchem disease, included facial palsy in all, various degrees of hearing impairment in 14/15 patients (Fig. 3e), symptoms of increased intracranial pressure in 3/15 patients, decreased sense of smell in 2/15 patients while none had visual impairment [71]. Different from patients with sclerosteosis, those with van Buchem disease have normal height and no digit abnormalities (Table 1). The clinical course of the disease stabilized in adulthood and no patient reported symptoms related to other organs such as heart, lungs, urogenital or gastrointestinal tract. Skeletal radiographs and CTs showed changes very similar to those of patients with sclerosteosis and BMD was greatly increased at both lumbar spine the hip with Z-scores ranging between +5.0 and +12.0. Serum P1NP values declined with age, as in patients with sclerosteosis, but remained either elevated or close to the upper limit of normal in adults. Laboratory investigations revealed no abnormalities in hematology or mineral metabolism. In a few bone biopsies from patients with van Buchem disease increased bone formation was documented (Fig. 3f) and lack of sclerostin expression in osteocytes. However, in one biopsy a weak sclerostin signal by immunological staining was observed.

**Table 1:** Similarities and differences of Sclerosteosis and van Buchem disease

Characteristic	Sclerosteosis	van Buchem disease
Genetic defect	Mutation SOST	52-kb deletion SOST
Inheritance	Autosomal recessive	Autosomal recessive
Stature	Tall	Normal
Syndactyly	Common	Absent
Facial palsy	Common	Common
Hearing loss	Common	Common
Increased ICP	Common	Uncommon
BMD	Increased	Increased
Serum Sclerostin	Undetectable	Very low

*ICP* intracranial pressure, *BMD* bone mineral density

### The cause

In patients with van Buchem disease, there were no mutations in the *SOST* gene but a 52-kb homozygous noncoding deletion 35 kb downstream of the *SOST* gene was identified [72, 73]. The deleted region contains a regulatory element particularly important for the gene transcription in bone but is not required for its embryonic transcription. These observations may explain the similar bone phenotypes of van Buchem disease and sclerosteosis and the absence of digit abnormalities in patients with van Buchem disease. In mice targeted deletion of ECR5, a bone enhancer located in the van Buchem deletion, increased bone mass and bone formation rates in trabecular bone with no effect on bone resorption and improved bone structure [74]. These changes were less pronounced than in *Sost* knock-out mice a finding consistent, according to authors, with the milder phenotype of van Buchem disease.

### The patient

The patient, a male of Dutch origin, presented at the age of 3 years with facial palsy and developed progressive deafness requiring a hearing aid by the age of 10 years followed by bilateral bone-anchored hearing aids [75]. He had a large skull and mandible but no abnormalities of hands or digits. He has been otherwise well with normal growth development. He was tall for age (above the 90th percentile, but both parents were also tall). He had three, phenotypically normal, sisters. During 15-year follow-up he had no signs or symptoms from other organs, and blood pressure was normal. Hematology and biochemistry, including parameters of mineral metabolism, demonstrated no abnormalities over the years. Skeletal radiographs showed thickening of the calvarium, the base of the skull and of the long bones and sclerosis of the vertebrae. Bone mineral density (BMD) of the spine and hip were markedly increased (*Z*-score +7.2). Spine BMD of the parents was also on the high side; the mother had a *Z*-score of +0.98 and the father of +0.85. Biochemical markers of bone turnover were always increased compared to normal values for age, but followed a normal pattern of change with a further increase during the growth spurt and a progressive decline thereafter, although never reaching the normal range. The diagnosis of van Buchem disease was confirmed by the finding of a 52-kb homozygous deletion 35 kb downstream of the *SOST* gene on chromosome 17q12-q21. Both parents were carriers of the disease.

### Sclerostin inhibitors

The restricted expression of sclerostin to the skeleton and the lack of abnormalities in organs other than the skeleton in patients with sclerostin deficiency made this protein an attractive target for the development of a new bone forming therapy for the management of osteoporosis. This approach was further supported by the gene-dose effect suggested by findings in heterozygous carriers of sclerosteosis who demonstrated decreased serum sclerostin levels associated with increased levels of P1NP and high normal or increased BMD without any clinical symptoms, signs, or complications of sclerosteosis [53, 54]. The most frequently used sclerostin inhibitor in preclinical and clinical studies is romosozumab (AMG 785, a humanized monoclonal antibody) while blosozumab (humanized monoclonal antibody) has also been used in clinical studies.

#### Animal studies

In OVX rats and nonhuman primates, injections of sclerostin antibody (Scl-Ab) increased dramatically the rate of bone formation at all skeletal envelopes and bone mass and strength at multiple sites [76–78]. Importantly, the majority of new bone formation was modeling-based occurring at quiescent surfaces, a true anabolic response [79]. The increase in bone formation induced by Scl-Ab was not associated with an increase in bone resorption. Instead, a decrease of osteoclast surface was observed, suggesting a functional uncoupling between bone formation and bone resorption. Despite increases of 54 % in bone volume, matrix mineralization was not affected [80]. The effect of sclerostin inhibition on bone formation was reversible upon discontinuation of treatment.

#### Human studies

**Fig. 5:**
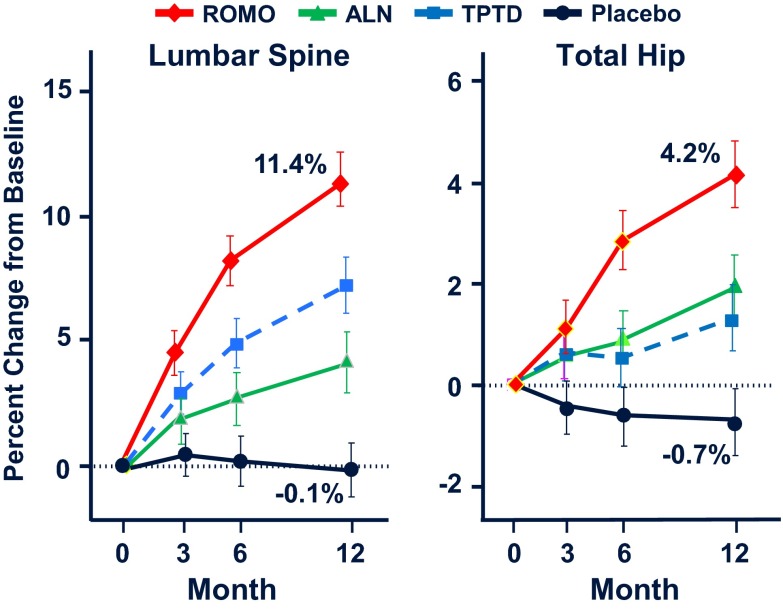
Percent changes of lumbar spine and total hip BMD during treatment of women with low bone mass with romosozumab (ROMO) 210 mg once-monthly sc, teriparatide (TPTD) 20 μg daily sc, alendronate (ALN) 70 mg once-weekly orally, or placebo (from [83])

In phase 1 human studies, administration of single or multiple doses of romosozumab and blosozumab increased bone formation markers and decreased bone resorption markers associated with significant increases in BMD [81, 82]. A phase 2 clinical trial of the efficacy and tolerability of romosozumab in postmenopausal women with low bone mass compared different doses and dosing intervals of subcutaneous injections of romosozumab with placebo, oral alendronate 70 mg weekly, and subcutaneous teriparatide 20 μg daily [83]. Romosozumab, 210 mg once-monthly sc, increased BMD at the spine (11.3 %), total hip (4.1 %), and femoral neck (3.7 %) (Fig. 5). These increases were significantly higher than those observed in women treated with either alendronate or teriparatide. No differences in BMD of the distal third of the radius were observed between any of the studied groups. Adverse events were similar between groups except for mild reactions at the injection sites of romosozumab. Continuation of romosozumab treatment for a second year was associated with further increases in spine and total hip BMD to total gains of 15.7 and 6.0 %, respectively, while serum P1NP and CTX levels remained below baseline values [84]. Women were then randomized to receive denosumab or placebo for an additional year. Women who transitioned to denosumab continued to accrue BMD at a rate similar to that with romosozumab during the second year, while in those who transitioned to placebo BMD returned towards pretreatment levels; similar results were reported after discontinuation of one year blosozumab treatment [85].

**Fig. 6:**
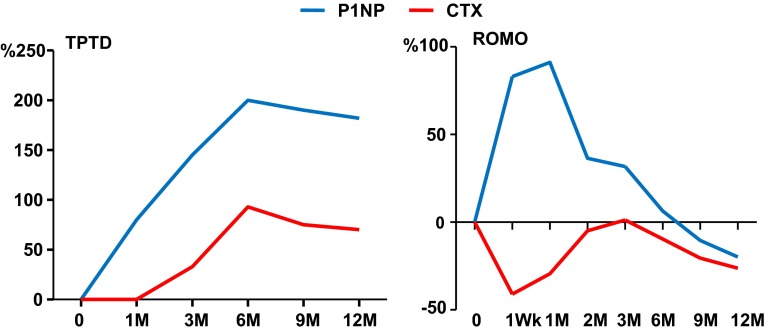
Schematic representation of changes in levels of serum biochemical markers of bone formation (P1NP) and bone resorption (CTX) during treatment with subcutaneous injections of either teriparatide (TPTD, 20 μg daily) or romosozumab (ROMO, 210 mg once monthly) for 1 year (from [86])

Kinetics of biochemical markers of bone turnover during treatment with romosozumab were intriguing and different from those observed during treatment of patients with other antiosteoporotic agents (Fig. 6) [86]. There was an early rapid increase in bone formation markers followed by a progressive decline with time, which was not due to the development of neutralizing antibodies. The effect of sclerostin inhibition on bone formation markers was further associated with a decrease of bone resorption markers, possibly through an inhibitory effect of the antibody on the production of RANKL/OPG by the osteocytes [64]. Treatment prolongation, however, appears to modestly reduce bone resorption but also bone turnover. It is postulated that while romosozumab acts as pure anabolic agent in the beginning of treatment, prolonged administration results in mild inhibition of bone resorption and reduction of the remodeling space. The mechanism of this response has not yet been clarified. Phase 3 clinical studies are currently investigating the anti-fracture efficacy and tolerability of romosozumab versus placebo or bisphosphonate in patients with osteoporosis (www.clinicaltrials.gov) and the first results are expected in 2016.

### Comment

Outcomes of studies of human and animal sclerostin deficiency are remarkably similar and were reproduced in preclinical and clinical studies with sclerostin inhibitors (Table 2). Thus, the human disease provided not only the model for the identification of sclerostin and its importance in bone remodeling, but could also predict the response of patients with osteoporosis to sclerostin inhibitors. Increases in BMD with romosozumab treatment in animals and humans were progressive but the slope of the increase changed with time; a finding in line with the observations of patients with sclerostin deficiency in whom stabilization of the disease was invariably observed after the third decade of life. Together these findings suggest that treatment of patients with osteoporosis with sclerostin inhibitors will be of limited duration and will form part of a treatment strategy rather than monotherapy, particularly for patients with severe osteoporosis.

**Table 2:** Consistency of findings in patients with sclerostin deficiency and treatment of animals and humans with sclerostin antibody

	Sclerosteosis	Scl-Ab animals	Scl-Ab humans
Bone mass	Increased	Increased	Increased
Bone strength	Increased	Increased	Increased^a^
Bone formation	Increased	Increased	Increased^b^
Bone resorption	Normal or decreased	Decreased	Decreased^b^
Anabolic response	Decreases with age	Declines with time	Declines with time

^a^Assessed by finite element analysis

^b^Assessed by markers of bone turnover

## Conclusions

Study of the genetics and pathophysiology of three very rare skeletal disorders provided new effective interventions for osteoporosis with different mechanisms of action. These, together with other agents, in combination or sequentially, will form, in our opinion, the basis of treatment of the individual patient with osteoporosis in the future, as it occurs in other chronic diseases. Crucial for the successful application of these new treatments for clinical practice will be their tolerability profile that still needs to be fully established.
